# Development of DArT-based PCR markers for selecting drought-tolerant spring barley

**DOI:** 10.1007/s13353-015-0273-x

**Published:** 2015-02-26

**Authors:** Anna Fiust, Marcin Rapacz, Magdalena Wójcik-Jagła, Mirosław Tyrka

**Affiliations:** 1 Department of Plant Physiology, University of Agriculture in Kraków, ul. Podłużna 3, 30-239 Kraków, Poland; 2 Department of Biochemistry and Biotechnology, Rzeszow University of Technology, Albigowa 472, 37-122 Albigowa, Poland

**Keywords:** Barley, Drought tolerance, Marker conversion, DArT, STS, SSR

## Abstract

The tolerance of spring barley (*Hordeum vulgare* L.) cultivars to spring drought is an important agronomic trait affecting crop yield and quality in Poland. Therefore, breeders require new molecular markers to select plants with lower spring drought susceptibility. With the advent of genomic selection technology, simple molecular tools may still be applicable to screen material for markers of the most important traits and in-depth genome scanning. In previous studies, diversity arrays technology (DArT)-based genetic maps were constructed for F_2_ populations of Polish fodder and malt barley elite breeding lines, and 15 and 18 quantitative trait loci (QTLs) related to spring drought tolerance were identified, respectively. In this paper, we show the results of a conversion of 30 DArT markers corresponding to 11 QTLs into simple sequence repeat (SSR) and sequence tagged site (STS) markers. Twenty-two polymorphic markers were obtained, including 13 DArT-based SSRs. Additionally, 31 SSR markers, located in close proximity to the DArT markers, were selected from the GrainGenes database and tested. Further analyses of 24 advanced breeding lines with different drought tolerances confirmed that five out of the 30 converted markers, as well as three out of the 31 additional SSR markers, were effective in marker-assisted selection for drought tolerance. The possible function of clones related to these markers in drought tolerance is discussed.

**Electronic supplementary material:**

The online version of this article (doi:10.1007/s13353-015-0273-x) contains supplementary material, which is available to authorized users.

## Introduction

A goal of plant breeding research is the discovery of methods that allow for the selection of genotypes characterized by higher levels of tolerance to stress factors (Vinocur and Altman 2005). To improve stress tolerance in economically important crops, both conventional plant selection methods and marker-assisted methods have been used (Cattivelli et al. 2008). Molecular biological tools are frequently used to identify the genetic background of phenotypic and physiological characteristics of plants exposed to stress factors (Mir et al. 2012). One of the most important methods is marker-assisted selection (MAS), which aims to identify molecular markers strongly associated with a particular trait. MAS combines the knowledge about a genotype and a phenotype of the analyzed plants, and improves the efficiency of plant selection in their early developmental stage (seedlings), thus reducing the breeding time and cost. The advantage of MAS is that it increases the efficiency of conventional selection methods and offers the possibility to identify genotypes whose phenotypic characteristics are the results of the simultaneous interaction of multiple genes. The use of molecular markers in the breeding process increases the selection sensitivity, enables the identification of plants with desired traits, and is sensitive to the environment (Collard et al. 2005).

Drought is a widespread phenomenon that occurs naturally worldwide (Cattivelli et al. 2008). Despite the fact that Poland is located in the temperate climate zone, for hundreds of years, it has been threatened by drought. Data from the last 25 years indicated an increasing frequency of spring drought events in Poland (Kożuchowski and Degirmendžić 2005). The problem occurs particularly in the central, northwestern, and central-eastern part of the country. Drought results in increasing differences between the size of the maximum expected and actual plant yield restricted by climatic and soil conditions. Spring drought is a serious problem in Poland that ultimately leads to the deterioration of the national economy and industry by reducing the quality and quantity of crop yield (Martyniak et al. 2007; Kalbarczyk 2010).

Spring barley (*Hordeum vulgare* L.) is one of the most popular model organisms used for genetic and physiological studies of grasses, and has been used extensively in research on drought tolerance. Drought tolerance in barley is complex; therefore, research aimed at improving this trait is difficult (von Korff et al. 2008). Barley is a diploid annual plant, with a short life cycle and a relatively small (5.1 Gb), recently sequenced genome comprising seven chromosomes (International Barley Genome Sequencing Consortium 2012). Barley is widely adaptable to different environmental conditions, and has huge genetic, morphological, and physiological diversity. Its economic importance meant that it was the first plant in which molecular markers were applied in practice (Forster et al. 2000).

When searching for an efficient molecular marker system for drought tolerance, strong genotype and quantitative trait locus (QTL) × environment interactions should be taken into consideration (Cattivelli et al. 2008). Drought tolerance is strongly affected by the local environment; therefore, different site-specific physiological and molecular adaptations are required, generating constraints for the general exploitation of QTLs for effective MAS. Considerable variations in drought tolerance were observed in the spring barley accessions taken from current breeding programs of Polish companies. Different physiological adaptations were triggered by an incidental selection towards drought tolerance in some of these materials bred for malting quality (Rapacz et al. 2010). Genetic mapping of the loci involved in drought tolerance in the malting and fodder barley bred in Poland in separate breeding programs revealed 18 and 15 QTLs, respectively, for nine physiological traits desired in drought-tolerant plants under Polish conditions. The main QTLs were located on chromosomes 4H, 5H, and 7H (Wójcik-Jagła et al. 2013).

Efficient localization of QLTs in crosses between germplasms from on-going breeding programs was possible because of the use of high-throughput phenotyping and genotyping methods. Microarray-based diversity arrays technology (DArT) markers are commonly used in plant genomics (Appleby et al. 2009), and the system has been integrated recently with rapidly developing genotyping by sequencing (Poland et al. 2012). DArT allows the simultaneous analysis of hundreds or even thousands of polymorphisms generated by genome rearrangements, generating a genetic profile of a plant without knowing its DNA sequence (Zhang et al. 2009). However, DArT markers are arbitrary, dominant, and expensive to use. Although high-throughput marker systems provide advanced tools for genomic selection, it is economically justified to use methods that preselect the tested materials. Conversion of DArT markers into classical polymerase chain reaction (PCR)-based technology opens up the possibility of low-cost testing of selected main QTLs. The most common PCR markers used in MAS are simple sequence repeats (SSRs) (Zhou et al. 2003) and sequence tagged sites (STSs) (Collard and Mackill 2008). Locus-specific SSR markers have been identified in many species, including barley (Agarwal et al. 2008). SSRs are short motifs (1–6 nucleotides) that are tandemly repeated several times, with a high degree of polymorphism. Microsatellites have multi-allelic and co-dominant characters, and their identification requires only a small amount of plant material (Powell et al. 1996a, b; Gupta and Varshney 2000).

Although the DArT system is an effective tool for the prompt identification of QTLs, the adaptation of selected markers to PCR technology would be, for economic reasons and data transferability, more suitable and beneficial for use in the MAS system. Thus, the aim of the current study was to verify the suitability of barley DArT markers for conversion into PCR-based markers and to verify the selection value of the newly developed markers for drought tolerance.

## Materials and methods

### In silico analysis

Two genetic linkage maps for malting and fodder genotypes of Polish spring barley were created using the DArT marker system. QTL regions for physiological parameters associated with the drought response were described in detail in a previous publication (Wójcik-Jagła et al. 2013) and were used to select DArT markers for conversion. Among the 33 QTLs reported, 11 QTLs with the highest percentage of explained variation (>10 %), high additive effects, and the greatest physiological importance were chosen. Additionally, seven regions significant in the preliminary simple marker regression analysis were selected. Thirty non-redundant clone sequences out of 53 DArT markers selected by these criteria were obtained from the Diversity Arrays network (http://www.diversityarrays.com/).

All 30 DArT clone sequences obtained in the previous step were tested by MISA software to find microsatellite repeats (Thiel et al. 2003). To identify as many potentially polymorphic regions as possible, the microsatellite defining criteria were restricted to the following (size of motifs/minimal number of repeats): (1/8), (2/5), (3/4), (4/4), (5/3), and (6/2). The maximum distance between imperfect microsatellites was set to 100 bp. Sequences lacking microsatellite motifs were used to identify STS markers by PCR reactions with specially designed primers (Table S1). Additionally, for some chromosome regions, 31 SSR markers selected from barley genetic maps constructed with DArT and SSR markers in the GrainGenes database were tested (Table S2). The sequences of SSR markers were obtained from the GrainGenes and HarvEST:Barley databases (http://www.barleybase.org/ and http://harvest.ucr.edu/). The PCR primers were designed using Primer3 software (v. 0.4.0).

To discern original DArTs, a “T” prefix was added to the name of the DArT marker adapted into the PCR system. The markers were assigned to bin locations based on the barley maps (Wenzl et al. 2006; Aghnoum et al. 2010; Wójcik-Jagła et al. 2013; König et al. 2014) and the data from http://barleygenomics.wsu.edu/. The physical locations of DArT clones were established with respect to “Morex” (an assembly of whole-genome shotgun sequence from barley) using the IPK Barley BLAST Server (http://webblast.ipk-gatersleben.de/barley/). Polymorphism information content (PIC) values were calculated as in Tyrka et al. (2008). The functional annotation of sequences was performed using the Blast2GO software (Conesa et al. 2005).

### Marker verification

#### Plant material

In the first step, the markers were tested in the breeding lines used to create the mapping populations (STH369 and MOB12055 for fodder barley, and STH836 and STH754 for malting barley). Nineteen selected markers with good technical quality of detection and polymorphism between parental lines were used in the next step to analyze the mapping populations of malting and fodder barley, and to test a set of 24 additional accessions with phenotypic characteristics. These 24 genotypes included spring malting barleys (STH1146, MOB6562, STH917, STH779, MOB9609, STH1036, MOB10740, MOB11558, MOB7890, MOB10654, MOB11723, and MOB11990) and fodder barleys (STH1067, MOB11803, MOB5735, STH906, MOB11728, STH828, STH1034, STH1112, STH1113, MOB7009, STH915, and STH858). All the lines, except for STH1113, were characterized for their physiological response to drought in a paper by Rapacz et al. (2010).

#### Sampling and PCR

The third leaves of plants grown in greenhouse conditions were used for DNA isolation (DNeasy Plant Mini Kit, Qiagen, Hilden, Germany) (Wójcik-Jagła et al. 2013).

Polymorphisms of the markers were identified using PCR reactions and electrophoresis (Wang et al. 2003). DNA was amplified in a GeneAmp® PCR System 9700 (Applied Biosystems, Foster City, CA, USA). PCR parameters were established individually, depending on the primer melting temperature, reports selected from the literature, and the GrainGenes database (Tables S1 and S2). The PCR reaction volume (20 μL) contained 1× PCR buffer (Fermentas, Vilnius, Lithuania), 250 nM of each primer, 2.5 mM MgCl_2_, 200 mM dNTP, 0.4 U Taq polymerase (Fermentas), 20 ng of DNA, and 400 μM spermidine (Sigma-Aldrich, St. Louis, MO, USA).

#### Agarose and polyacrylamide gel electrophoresis

Marker polymorphisms were visualized after electrophoresis through a 1.5 % agarose gel (ag × arose dissolved in 1× TBE buffer with 0.015 μL ethidium bromide mL^−1^) on an OWL AG electrophoresis system (Thermo Scientific, Waltham, MA, USA). Each sample for electrophoresis contained 6.5 μL sterile H_2_O, 3.5 μL of loading dye buffer (6× DNA Loading Dye, Fermentas), and 2 μL of PCR product. A DNA ladder (GeneRuler 100 bp DNA Ladder) was used as a product length indicator. The electrophoretic parameters were 5 V/cm, 3.5 mA/cm, and 0.4 W/cm. Gel visualization was carried out twice, after 1 and 2.5 h (Molecular Imager® ChemiDoc™ XRS System, BioRad).

Ambiguous results were analyzed again in denaturing conditions using a denaturing sequencing gel. The gel (96 mL) contained 60.8 mL 5 M urea, 9.6 mL 10× TBE buffer, 9.6 mL 40 % acrylamide with bisacrylamide (39:1), 150 μL APS, and 40 μL TEMED. The gels were subject to 30 min of pre-electrophoresis, after which DNA samples were denatured (95 °C, 5 min, 0 °C, 3 min) with formamide dye (in 1:1 volume) and loaded onto the gel in a 1.2-μL volume. The electrophoretic parameters were 1 h 15 min, 45 V/cm, 1.3 W/cm, 2.2 mA/cm. Visualization was carried out using the silver-staining method (Chalhoub et al. 1997).

#### Verification of marker efficiency

A detailed description of the phenotyping process and its results has been published previously (Rapacz et al. 2010). In general, the results of phenotyping comprised the stress indices [SI = (d/w) × 100 %, w : parameter value in a well-watered plant, d: parameter value after drought treatment] calculated for the physiological parameters. Drought induced changes in the following parameters: electrolyte leakage from leaf tissues (EL), leaf water content (WC), CO_2_ net assimilation rate (P_n_), transpiration rate (Tr), as well as different parameters of chlorophyll fluorescence describing: (1) the quantum yield of electron transport at PSII (φPSII); (2) the quantum efficiency of energy transfer between PSII antennas and reaction centers (F_v_/F_m_); (3) the overall performance index of PSII calculated for equal absorption (PI); (4) the flux of the energy absorbed in PSII antennas per leaf cross-section (CS): ABS/CS; (5) the flux of the energy trapped in PSII reaction centers (TRo/CS); (6) the flux of the energy used for electron transport (ETo/CS); (7) the energy dissipated from PSII (DIo/CS); and (8) the maximum number of active PSII reaction centers (RC/CSm). The rate of PSII quantum efficiency related to the quantum efficiency of CO_2_ assimilation, φPSII/φCO_2_, was also calculated.

The results of genotyping were compared with those of phenotyping using Spearman’s correlation coefficient calculated between the presence of a marker and the SI value for the physiological trait of the linked QTL, after converting the results from allelic into the 0–1 format. Additionally, statistical significance of the difference between drought susceptibility index (DSI) mean values in the plants containing different allelic variants of the marker was tested using the Mann–Whitney *U* test. In the case of markers characterized by the presence of different sized products, each pairwise comparison was performed. All the statistical analyses were done using STATISTICA 10.0 software (StatSoft, Tulsa, OK, USA).

## Results

### Searching for DArT clone sequences for QTLs related to drought tolerance

In both populations, the same criteria were used when choosing markers for further analysis. The basic criterion was association with a QTL, as previously published by Wójcik-Jagła et al. (2013). Additionally, markers that were highly correlated (*r* ^2^ > 0.5) with drought susceptibility indices for physiological parameters of the highest importance for drought tolerance were included. These parameters were EL, WC Pn, and φPSII/φCO_2_. For the malting barley population, nine chromosome regions were preliminarily selected to develop PCR-based markers. No sequence information for DArT bPb-20617 from the region of QPSII-CO2.sthm-6H was available and the number of regions was reduced. No sequences were available for the regions of interest on 1H (bPb-0915), 2H (bPb-2948), 3H (bPb-2394, bPb-39380), 6H (bPb-3554), and 7H (bPb-3020, bPb-8043). Ultimately, 16 non-redundant clone sequences for 27 DArT markers were selected for a conversion to STSs (Table 1).

**Table 1:** Chromosome regions of malting barley selected to saturate with molecular markers. ^×^ – markers tested on a validation set of 24 lines; M – monomorphic markers; A – markers polymorphic on agarose gel; P – markers polymorphic in sequencing polyacrylamide gel; H – markers heterozygous within parents. *QTLs according to Wójcik-Jagła et al. (2013)

Chromosome .bin/QTL*	DArTs transferability	DArTs length (bp)	SSR motifs	SSR from ± 5 cM regions
*1H.1*-*2*	bPb-1312 P^×^	820	–	
*1H.6*	bPb-2175 M	518	(CTTGCG)_2_	Bmag876*, A^×^
	bPb-8884 A^×^	548	–	EBmac405, M
				Bmag872, M
				Bmag211, M
*2H.6*	bPb-2230 H	277	(CTC)_4_	Bmag692, M
*2H.8*	bPb-0858 P^×^	311	(T)_8_, (CAGTGG)_2_	
*2H.14 QDI.sthm*-*2H*	bPb-1967 P^×^	674	–	
*3H.6*	bPb-5351 M	867	(ATAACT)_2_	Bmag131, P
*QABS*-*CS.sthm*-*3H*	bPb-4645 M	985	(TAGTTA)_2_	Hvm44, M
*QET.sthm*-*3H*	bPt-2040 A^×^	1,132	–	Bmag136, P
*QPI.sthm*-*3H*	bPb-8110 M	1,077	(AAGAAA)_2_	Bmac209*, A^×^
				Bmag006, P
				Bmag603, P
*6H.2 QRC*-*CS.sthm*-*6H.1*	bPb-2672 M	636	–	Bmag500, H^×^
*7H.1*-*2*	bPb-7863 M	388	–	EBmag794, P
	bPb-5902 A	624	–	Bmag007, P
	bPb-6450 A^×^	310	–	EBmac713, H
				Hvm04*, A^×^
*7H.13*	bPb-0259 A	709	(ACTA)_5_	Bmag206, P
	bPb-6399 A^×^	693	(T)_8_	

For the fodder barley, seven chromosome regions from 2H, 3H, 5H, and 6H were chosen and 14 non-redundant sequences of the clones from 26 DArT markers were obtained (Table 2). No sequences were available for DArTs bPb-7881, bPb-2762, and bPb-7217 from the region 5H.6 with QTLs QWC.sth-5H.2, and QEL.sth-5H.2; therefore, this region was excluded from further analysis. The sequences of the DArT clones were also not found for the chromosome regions 2H.11 (bPb-2005), 2H.15 (bPb-2244, bPb-9673, and bPb-1415), 3H.7 (bPb-5586), 5H.4 (bPb-4135), and 6H.6-7 (bPb-8347).

**Table 2:** Chromosome regions of fodder barley selected to saturate with molecular markers. ^×^ – markers tested on a validation set of 24 lines; M – monomorphic markers; A – markers polymorphic on agarose gel; P – markers polymorphic in sequencing polyacrylamide gel; H – markers heterozygous within parents. *QTLs according to Wójcik-Jagła et al. (2013)

Chromosome .bin/QTL*	DArTs transferability	DArTs length (bp)	SSR motifs	SSR from ± 5 cM regions
*2H.11*	bPb-0994 A^×^	435	–	GBM1208, P
*QWC.sthf*-*2H*	bPb-7671 H	126	–	Bmac144, M
*2H.15*	bPb-1051 P^×^	585	(T)_9_	
*Qqp.sthf*-*2H*	bPb-4601 A	756	–	
*QPSII.sthf*-*2H*				
*3H.7*	bPb-7786 A^×^	372	(T)_9_	
*5H.4*	bPb-8589 P^×^	872	(AG)_5_	scssr02503, P^×^
*QWC.sthf*-*5H.1*	bPb-8556 P	694	(AG)_5_	
*QEL.sthf*-*5H.1*				
*6H.1*-*2*	bPb-2957 M	86	–	
	bPb-9645 A^×^	148	–	
*6H.6*-*7*	bPb-6735 A^×^	355	(AGTAGC)_2_	Bmag210, P
*QEL.sthf*-*6H.1*	bPb-3230 M	334	(ATGTAG)_2_	scssr02093, P
	bPb-6721 A^×^	879	(GGT)_4_N_19_(GATTTG)_2_	Bmac047b, P
	bPb-4369 A	833	(ACAAAT)_2_,(AAATAT)_2_ (TCCAAA)_2_N_20_(CAC)_4_	Bmag867, P
	bPb-3773 A	353	(TGCTAC)_2_	GBM1400, M
				Bmag003, P
				Bmag378, M
				HvLOX, P
				Bmag613, P
				GBMS180, P
				EBmac602, P

### Efficiency of DArT markers conversion to PCR-ready markers

The analysis performed on the parental genotypes of the malting population allowed us to reject 14 out of 33 analyzed sequences: 11 were monomorphic and three showed heterozygosity of the parents of the mapping population. Nine markers were suitable for agarose gel electrophoresis and ten markers, the products of which had to be visualized using a denaturing polyacrylamide gel electrophoresis, were chosen for the malting barley (Table 1). For the fodder barley, we rejected six out of the 28 analyzed markers, leaving 22 for further analysis (eight in agarose gels, 14 in polyacrylamide gels). The number of markers to be tested was restricted mostly by the lack of polymorphism between the parents of the mapping populations: 19 preferably co-dominant markers ready to analyze in agarose gels were selected (Table 3). Localization of the markers was based on genetic maps and referred to the sequenced genome of the cultivar “Morex”. Precise localization of the markers was considered crucial for the comparison with already known loci affecting drought tolerance. Analyses of the selected markers within the fodder and malting mapping populations (among 183 specimens) confirmed the markers’ polymorphisms and the data were integrated with a published genetic map (Wójcik-Jagła et al. 2013). Three selected regions, 2H14-15, 3H.6-7, and 6H.1-2, were common for both of the studied mapping populations.

**Table 3:** Bioinformatics characterization of new drought tolerance markers in barley. D – dominant; CD – co-dominant type of marker. Localization in cM on the Morex genome sequence, number of hits giving a more significant score, and the E-value is given in brackets

Marker	PIC	Chromosome, BIN	Marker type	Barley BLAST position (cM)	Blastx annotation (similarity mean)
TbPb-1312	0.454	1H.1-2	D	1HS/4.1;5.7	Receptor-like kinase (95.1 %)
TbPb-8884	0.444	1H.6	CD	(2)1H/48.1	cop9 signalosome complex subunit 1-like (82.5 %)
Bmag876	0.444	1H.6	CD	–	–
TbPb-0858	0.478	2H.8	CD	2HL/81.8	Hypothetical protein F775_24911 (79.0 %)
TbPb-1967	0.469	2H.14	CD	2HL/118.0	–
TbPb-0994	0.375	2H.11	D	2HL/107.8	–
TbPb-1051b	0.278	2H.15	D	2HL/141.6	Zinc finger ccch domain-containing protein 17 (57.6 %)
TbPb-2040b	0.278	3H.6	D	3HL/66.5	Rp1-like protein (57.8 %)
Bmac209	0.492	3H.6	CD	–	–
TbPb-7786	0.499	3H.7	D	3HL/59.0	Jekyll protein precursor
scssr02503	0.531	5H.4	CD	5HS/42.8	Auxin-responsive protein iaa30-like (82.6 %)
TbPb-8589	0.454	5H.4	D	5HS/43.8	–
TbPb-9645	0.444	6H.1-2	D	(1)6HS/53.1	–
Bmag500	0.605	6H.2	CD	–	–
TbPb-6735	0.413	6H.7	CD	6HL/60.0	–
TbPb-6721	0.413	6H.7	D	(7)6HL/53.6	–
TbPb-6450	0.413	7H.1	D	(1)7HS/13.6	–
HVM04	0.469	7H.2	CD	7HS/13.9	Granule-bound starch partial (84.7 %)
TbPb-6399	0.486	7H.13	D	7HL/140.7	Disease resistance protein rga1 (65.2 %)

### Bioinformatics characteristics of DArT clones

To improve the efficiency of primer design, the sequences of the DArT clones were screened for microsatellite motifs (Tables 1 and 2). The analysis was carried out using the MISA software and identified SSR motifs in 18 out of 31 analyzed sequences, with a total length of 17,397 bp. This gives an approximate frequency of one potential microsatellite motif per 1,000 bp of random DArT sequences. However, Spearman correlation of the clone length with the number of microsatellites was not significant (0.206, *p* = 0.266). Twenty different microsatellite repeats were identified. The most abundant were hexanucleotide and single nucleotide repeats, accounting for 60 % and 16 % of motifs, respectively. Two-, three-, and four-nucleotide motifs were less frequently observed (12 %, 8 %, and 4 %, respectively). Excluding imperfect microsatellites, two of the analyzed sequences (bPb-0858 and bPb-4369) contained double SSR motifs (Tables 1 and 2). The presence of a microsatellite motif was not significantly correlated with marker polymorphism (*r* = −0.031, *p* = 0.87).

The chromosomal locations of the DArT sequences were verified by BLAST searching against the sequenced genome of barley cv. “Morex” (http://webblast.ipk-gatersleben.de/barley/, Table 3). A screen of 15 sequenced DArT clones revealed that 12 sequence locations were concurrent with bin locations, while for bPb-8884, bPb-9645, and bPb-6721, the source sequence used for primer design annealed to multiple locations, and the target location did not have the highest significance. Functional annotation of the analyzed clones was significant for two DArT clones and two available SSR markers, and the sequences of bPb-8884 and scssr02503 contained domains of response to stress (GO:0006950) and to abiotic stimulus (GO:0009628).

### Verification of marker efficiency in the selection of barley genotypes towards drought tolerance

Nineteen PCR markers selected in the previous step showed polymorphisms in the 24 studied barley accessions. In this experiment, we omitted the extremely drought-tolerant and drought-susceptible genotypes used as the parents for the mapping populations. The correlation between the presence of a particular allele and the physiological traits connected with drought tolerance, as well as marker selection effectiveness (a difference in DSI values between groups of accessions containing different alleles) showed that the set of the tested sequences contained markers that could be used effectively in the selection of drought-tolerant genotypes (Table 4).

**Table 4:** Selection value of drought tolerance markers tested in the group of 24 malting and fodder barley advanced breeding lines. Only markers showing significant Spearman correlation coefficients (*r*_S_) with the phenotypic parameters are shown. The mean value (%) of the relative drought susceptibility index (DSI, the relative value in drought when compared to the control conditions) value in the candivars with product observed (product+) in relation to the value in the candivars with product not observed (product−) is also presented. Significance of the differences (*U* test) between product+ and product− candivars was marked: *p < 0.05, **p < 0.01, and ***p < 0.005, respectively

Marker	Source population	Phenotypic parameter (DSI)	Malting		Fodder	
			*r*_S_	product+/product− (%)	*r*_S_	product+/product− (%)
TbPb-0994_330_	FO	φPSII/φCO_2_			0.57	192.5*
		DIo/CS	0.84	109.9***		
		WC	0.66	116.4*		
		F_v_/F_m_			−0.63	82.5*
		φPSII			0.67	74.1*
TbPb-1967_203_	MA	φPSII/φCO_2_	0.75	142.3**	−0.58	61.3*
		P_n_	0.64	146.2*		
		Tr			−0.62	77.2*
		F_v_/F_m_			0.67	113.3*
TbPb-1967_200_		φPSII/φCO_2_	−0.75	70.3**	0.58	163.1*
		P_n_	−0.64	68.4*		
		Tr			0.62	129.6*
		F_v_/F_m_			−0.67	88.3*
Bmac209_200_	MA	F_v_/F_m_			0.72	111.4**
		φPSII			0.72	115.9**
Bmac209_180_		F_v_/F_m_			−0.72	89.8**
		φPSII			−0.72	86.3**
Bmag500_150_	MA	φPSII			−0.65	90.5*
		EL	−0.67	58.0*		
Bmag500_120_		φPSII/φCO_2_	0.66	137.8*		
		Tr			0.75	110.8***
		ABS/CS			0.75	108.5***
		TRo/CS			0.75	110.8***
		RC/CS			0.59	110.0*
scssr02503_110_	FO	EL	−0.78	53.1***		
TbPb-6399_170_	MA	Tr	0.65	128.0*		
		φPSII	0.65	109.8*		
		F_v_/F_m_	0.77	109.5**		
TbPb-9645_480_	FO	ETo/CS			0.58	113.8*
TbPb-0858_300_	MA	WC			−0.66	93.0*
TbPb-0858_280_		WC			0.58	106.2*

According to the statistical analysis performed for all 24 malting and fodder barley candivars (i.e., promising new candidate cultivars) used in the experiments, 12 alleles of eight analyzed markers showed significant values for Spearman’s correlation coefficient, ranging from 0.84 to −0.78, when the best correlating phenotypic characters were taken into consideration (Table 4).

Among seven alleles of the six markers significantly correlated with the physiological traits in the malting barleys, the highest values of Spearman’s coefficients were observed for bPb-0994 (positive correlation with DIo/CS) and scssr02503 (negative correlation with EL). In the case of scssr02503, the observed differences between candivars in which the product was or was not observed (product+/product−) were also the highest when compared with other markers and DSIs. Additionally, five other alleles of three markers were significantly correlated with drought-induced changes in different aspects of PSII photochemical activity, whereas two alleles of marker bPb-1967, on chromosome 2H, significantly correlated with the drought effect in relation with PSII and CO_2_ quantum efficiency, as well as the net assimilation rate. The significant correlation of marker scssr02503 with the drought-induced changes in the physiological parameters describing electrolyte leakage, the correlation of marker bPb-6399 with maximum (F_v_/F_m_), and the actual (φPSII) quantum efficiency of PSII were observed for the malting barleys.

In the fodder-type barleys, statistically significant correlations with drought-induced changes in physiological parameters were observed for ten alleles of six markers, among which five alleles of three markers were unique in the discrimination of fodder barleys only. The highest value of Spearman’s correlation coefficient was observed for Bmag500 (with Tr, ABS/CS, and TRo/CS). In addition, six alleles of four markers within the fodder barleys correlated significantly with DSIs, a parameter associated with different aspects of the photochemical activity of PSII. bPb-0994 and both of the Bmac209 alleles were useful to discriminate a maximum (F_v_/F_m_) and actual (φPSII) quantum efficiency of PSII. For the two remaining markers, unique in the discrimination of drought tolerance among the fodder barleys (bPb-9645 and bPb-0858), only associations with WC and ETo/CS were observed, respectively (Table 4).

## Discussion

The identification of barley co-dominant and specific molecular markers based on the arbitrary dominant DArT markers offers an opportunity to use molecular markers effectively in barley breeding. The MAS system, which requires molecular markers to select plants with particular desired traits, makes it possible to increase breeding efficiency at a relatively low cost (Collard et al. 2005). Some DArT markers without SSR motifs were polymorphic, most likely reflecting changes in the primer binding site sequence. The co-dominant character of STS and microsatellite sequences, their high reproducibility, and their suitability for high throughput and automation, makes them technically simple to use (Semagn et al. 2006). Although we selected the markers using basic equipment, more advanced marker technologies involving next-generation sequencing should select markers representing single nucleotide polymorphisms. Among the different methods of single nucleotide polymorphism detection, single-strand conformational polymorphism, high-resolution melt, or cost-effective competitive allele-specific PCR (KASPar) markers should be considered as the methods of choice (Allen et al. 2011; Cortés et al. 2011). Furthermore, specific molecular marker applications enable researchers to examine more complicated epistatic effects and genotype × environment interactions.

There are many reports on the improvement of the genetic pool using molecular markers for traits with either simple or complex genetic control in barley and in other species (Mohan et al. 1997; Young 1999). However, there are few examples of the practical use of QTLs or the markers derived from them. One study showed the use of the molecular markers flanking two main QTLs for the malting quality of barley (Han et al. 1997). In the case of drought tolerance improvement, the reports of MAS application have been limited. In maize, genotypes with a lower degree of anthesis-silking interval (ASI) were selected using MAS, because of the negative correlation of this trait with grain yield under limited water conditions (Ribaut et al. 2002, 2004). Another example involved the transfer of a deep root system from a wild rice to a rice cultivar with a shallow root system (Courtois et al. 2003; Shen et al. 2001).

Despite numerous experiments involving QTL analysis, molecular markers useful for the selection of barleys have not been presented so far. This probably reflects the problem of adjusting the results obtained for “model crosses” of barley and the materials available in the breeding programs. F_2_ populations are commonly used as mapping populations to identify molecular markers. They are the offspring of F_1_ populations, obtained most frequently by crossing plants not related to the current breeding materials. Barley mapping populations for drought resistance are usually derived from crosses with, for example, wild barley *H. spontaneum* or primitive landraces, revealing genetic marker associations with site-of-origin ecogeographic factors and stress (Forster et al. 2000). This caused fundamental problems for the use of markers in the selection among modern genotypes. In the present study, the markers were derived from QTLs of physiological characteristics determined in mapping populations derived from crosses between spring barley entries chosen from among Polish breeding materials with contrasting drought tolerances (Rapacz et al. 2010; Wójcik-Jagła et al. 2013). Our markers were then tested successfully in the wider range of current breeding materials, which makes our strategy promising for generating working marker systems. Selecting current breeding materials at the very beginning of a marker search tends to be easier during the time of dynamic development of new genotyping and phenotyping methods, offering increasing sensitivity.

In our study, eight markers were identified that correlated with drought tolerance mechanisms important for the development of drought tolerance in the Central European climate.

A number of genes that are crucial in the determination of drought tolerance in barley, such as *ICE2* (inducer of CBF expression), showed no significant effects in our study. We found no effects for the region of *HVA1* (late embryogenesis abundant protein encoding gene) from chromosome 1H, mapped between the markers MWG706 (2.3 cM) and ABC257 (3.3 cM) of the DArT consensus map (Wenzl et al. 2006), corresponding to bin12. Similarly, *FRY1* and *SRG6* (hypothetical transcription factor) genes, found at 7H bin 4 and bin7, respectively (Malatrasi et al. 2002; Aghnoum et al. 2010), were not responsible for the differences in drought tolerance observed in our materials. The *ICE1* gene, mapped in the “Proctor” × “Nudinka” population at chromosome 7H and at a position of 82.1 cM (Tondelli et al. 2006), overlapped with the *SRG6* gene. *ICE2* was mapped on chromosome 3H (bin 13) of a Dicktoo × Morex barley mapping population at a position of 133 cM (Skinner et al. 2006). Instead, at least three regions overlapped in the malt and fodder barley populations. Thus, the regions 2H.14-15, 3H.6-7, and 6H.1-2 are of special interest.

Two of the analyzed markers, bPb-2040b and Bmac209, which correlated with *QABS*-*CS.sthm*, *QET.sthm*, and *QPI.sthm* QTLs (Wójcik-Jagła et al. 2013), identified on chromosome 3H bin6, also overlapped with the AWBma15 locus identified within OUM23 lxCI 3208-1 (Collins et al. 1996). In addition, bPb-6721 and bPb-6735, associated with *QEL.sthf*-*6H.1* QTL (Wójcik-Jagła et al. 2013), and found at chromosome 6H (bin7), had the same location as BCD340E and ksuD17, which correlated with the barley QTL *QSss.StMo* for salt tolerance at the seedling stage on chromosome 6H (Mano and Takeda 1997). Additionally, both markers were situated in the vicinity of marker ABG458, corresponding to barley QTL *QSsg.StMo* at bin6 on chromosome 6H (Qamar et al. 2008). Moreover, an effect was found from the region of dehydrin Dhn8 [late embryogenesis abundant (*LEA*) D11 proteins], which overlapped with the location of marker bPb-6735. The expression of *Dhn8* was reported to be associated with the low temperature and dehydration response in barley (Zhu et al. 2000; Choi et al. 2000). Five of the 15 remaining markers were found close to other loci, as published on the Barley Abiotic Consensus Map (http://wheat.pw.usda.gov). Markers bPb-8884 and Bmag876 at chromosome 1H bin6 were located close to the WG789B locus found at bin5 (1H)-7 (Rodriguez et al. 2006). Markers bPb-7786 and bPb-0994 were mapped close to WG405B and Rn5S1 (Kolchinsky et al. 1991). Among the markers tested within barley genotypes, bPb-0858 mapped at bin8 on chromosome 2H and showed high similarity to hypothetical protein F775_24911, which was localized between MWG557 (bin7) locus and ABC152D and His3C (bin9) on 2H. The mapped locations of the ten remaining markers showed no similarity to any identified barley locus. scssr02503 is an interesting marker because of its high negative selection value in discriminating the genotypes with plasma membrane integrity (EL) disturbed under drought, which is one of the most reliable parameters of drought tolerance in plants (Bajji et al. 2002). scssr02503 was correlated with QTLs *QWC.sthf*-*5H.2* and *QEL.sthf*-*5H.2* on chromosome 5H (Wójcik-Jagła et al. 2013). Functional annotations of this clone (Blast2GO software) showed the presence of domains responsible for stress response and a response to abiotic stimulus, and high similarity to the sequence of the Auxin-responsive protein iaa30-like (82.6 %). For the remaining sequences, similarities to different domains implicated in diverse biological processes were found. The highest similarity was observed to a receptor-like kinase involved in various signaling processes (95.1 %) for marker bPb-1312, and marker bPb-8884 was 82.5 % similar to the cop9 signalosome complex. Similarities to other domains related to disease response in plants such as Rp-1-like protein (57.8 %) and disease resistance protein rga1 (65.2 %) were observed for bPb-2040b and bPb-6399, respectively.

A particularly interesting marker among those described herein was bPb-1967, with two allelic variants “a” and “b” that showed positive or negative correlations, respectively, with a net assimilation rate decrease during drought. This marker was very efficient in the discriminating of genotypes according to their net photosynthesis rate decrease during drought, which is extremely important for maintaining a high growth rate and yield under water-deficit conditions (Lawlor and Cornic 2002).

In our study, only a small number of previously identified DArT markers were investigated. We chose those with the highest degree of variation explained by QTLs associated with a particular marker, located within QTLs of higher physiological importance, as well as those with a higher level of correlation with the loci and positions of genes important for drought tolerance. This reduction in the pool of analyzed markers made it impossible to determine whether there was any correlation between the efficiency of conversion and a particular QTL’s relevance.

The molecular markers obtained herein correlated with the traits responsible for different physiological effects of drought stress. Most of the markers correlated with the traits (or/and were linked with QTLs of traits) belonging to more than one of the following groups: plasma membrane integrity, water relations, gas exchange, and photochemical activity of PSII. This observation indicated that some regions of the barley genome are responsible for differences in more general drought response observed between the studied materials, as reported previously by Wójcik-Jagła et al. (2013). However, some markers were suitable for selection only in the malting or fodder barleys, but not in both. This may be an effect of the studied materials, where differences in drought tolerance were associated with different physiological characteristics of the malting and fodder barleys (Rapacz et al. 2010). Additionally, according to the same study, malting barleys were characterized by a higher drought tolerance level. Thus, molecular markers discussed herein cannot be excluded from the selection of drought-tolerant genes in a general sense; however, their effectiveness may be different depending on the drought tolerance level of the initial materials.

## Electronic supplementary material

Below are the links to the electronic supplementary material.

ESM 1 (DOCX 26 kb)

ESM 2 (DOCX 17 kb)
