# Supplementary material for: Development of DArT-based PCR markers for selecting drought-tolerant spring barley
Source: J Appl Genet. 2015 Feb 26;56(3):299–309. doi: 10.1007/s13353-015-0273-x (PMC4543407; doi:10.1007/s13353-015-0273-x)
Supplement: Supplementary file 1 — (DOCX 26 kb) [file 13353_2015_273_MOESM1_ESM.docx]

Development of DArT-based PCR markers for selecting drought tolerant spring barley

Journal of Applied Genetics

Anna Fiust, Marcin Rapacz (corresponding author), Magdalena Wójcik-Jagła, Mirosław Tyrka

University of Agriculture in Kraków, Department of Plant Physiology, ul. Podłużna 3, 30-239 Kraków, Poland**,** Email: rrrapacz@cyf-kr.edu.pl

Table S1. Sequences of PCR primers designed for 30 not-redundant DArT sequences from regions responsible for drought tolerance and annealing temperatures (Ta) used in PCR reaction.

| Marker | T_a_ (°C) | Primer sequences (5’ - 3’) | |
| --- | --- | --- | --- |
| TbPb-0259 | 55 | CAGATGAAATGACCGAGCAG | TCCGGAGCTAAAAGAGCAAG |
| TbPb-0858 | 58 | GGCAGGTACACCGCCACT | TCAGAGCACACGTATGCAGAT |
| TbPb-0994 | 52 | CCACCCCAATGTGTTCTCTC | TGCAGGCGAAAATTGTTGTA |
| TbPb-1051 | 55 | CGTCCCCATGATCCTTTTT | GCAGGCTATTTTGTGGCTTT |
| TbPb-1051b | 55 | CGGGAAGCTCTATCACTCGT | TGATATGTGCAGCGTCCATT |
| TbPb-1312 | 52 | TGAAACATCGAAACCCACAA | CTCCATTCCTCTGGCTATGC |
| TbPb-1967 | 55 | AGGTTTTCAAGCAGCTACGC | CAAGAAAGCAGATGGCACAA |
| TbPb-1967b | 55 | TTGCAGAAGGCGGATAATTC | TTTCGGGCACTGATTTCAAC |
| TbPb-2040 | 55 | CCTCAAGAGACAACGGCAAT | CGTCAGGTCTTTGTCGACTTC |
| TbPb-2040b | 55 | CCATAAAGTTAAGAATTTGCCTCA | GCAACTCACACCCCTTCTGT |
| TbPb-2175 | 55 | AAGCCAAAGCCTAACAGCAG | CAGTGCAGCATCATATTTGG |
| TbPb-2230 | 58 | GAAGACTCTTCAGAGATGGTGC | TGCAGCCAATTATAATAATGAGTT |
| TbPb-2672 | 55 | GGCGAACCTAATTCATTGGA | TCGGTGAACCACATTGGATA |
| TbPb-2957 | 55 | CATGATAAAAACCAAGATGAAATG | CATGTTCCCAGAGGACACG |
| TbPb-3230 | 55 | ACCATGCCACATGATGAGAA | TGCAGAGGGATATGGATTTCA |
| TbPb-3773 | 55 | GCAGCAAAGAAACACAGTTTG | TTCGGTCCTTCTTGCATACC |
| TbPb-4369 | 55 | TGCACCGCCATCAAGTAGTA | GTGGATTGTGAGGCCGATT |
| TbPb-4601 | 55 | CATGAGTCCTGTACCCAGCA | TTGCCTTTTAGCCGACTGTT |
| TbPb-4645 | 55 | TGAACAAACGGATACGTCCA | TGTGGAATGTTTTAGGTGCAA |
| TbPb-5351 | 55 | GGCAACTTTTGTTGCAAGATT | TGTGACAATTTGAAGTGACATGG |
| TbPb-5351b | 52 | GCAGCAGATGACTTTGTGGA | TGAAATGCTCAAGCAGGAAA |
| TbPb-5902 | 55 | ACAAGATTTAAGCCGCGTTG | TTGCATGCTAAACGTGAACC |
| TbPb-6399 | 55 | TGCACAGCCTAAAAGAATCG | TGTTGGCACAGCATGTTAGC |
| TbPb-6450 | 52 | ACGCCCAAGTCACAAATCTT | GGTCCAGTTCCTGTTCTTGG |
| TbPb-6721 | 55 | GGAAAAACAAAACTGAGGCAAA | GTGGATTGTGAGGCCGATT |
| TbPb-6735 | 52 | TCAGGCATCTGCAATTTTTG | TTCGGTCCTTCTTGCATACC |
| TbPb-7671 | 55 | CTGCTGGACGGCGATTTC | TGGGATACTTTTGATGTTATAGAAGAA |
| TbPb-7786 | 52 | GCTGGAGACTTGGAGGACAG | TGGTTATTACCACAACCAGA |
| TbPb-7863 | 55 | GTGTGAGCCGGTTTTCTTTC | TTGTGGTTGACAACAATCCAG |
| TbPb-8110 | 52 | CATTCATGCATTGGCATTTT | TGCAGAGGAAGTGTCCATGT |
| TbPb-8110b | 55 | CAAGCAGGAAGGACCCAATA | GAGCGGGTTCATGGATTC |
| TbPb-8556 | 55 | ACAGCACCTGCAATTCGTG | TCCTCCCTAGGTAAGCTCTCTG |
| TbPb-8589 | 55 | AGCTCTCTGTAGATCAGGTTGC | CGACAACGGGAATGGAAC |
| TbPb-8884 | 55 | CATGTGCAAACTGTCCCAAC | CTAGCAGCAGCAAGTGCATC |
| TbPb-8884b | 58 | AGTCCCGTGTCCGCATATAG | GGTTGAGCATTCTGGAGAGC |
| TbPb-9645 | 55 | CATGTCAAAAGCTATGGATGC | CTTGCCCTCTCTCGTCAAAC |
