# Supplementary material for: Development of DArT-based PCR markers for selecting drought-tolerant spring barley
Source: J Appl Genet. 2015 Feb 26;56(3):299–309. doi: 10.1007/s13353-015-0273-x (PMC4543407; doi:10.1007/s13353-015-0273-x)
Supplement: Supplementary file 2 — (DOCX 17 kb) [file 13353_2015_273_MOESM2_ESM.docx]

Development of DArT-based PCR markers for selecting drought tolerant spring barley

Journal of Applied Genetics

Anna Fiust, Marcin Rapacz (corresponding author), Magdalena Wójcik-Jagła, Mirosław Tyrka

University of Agriculture in Kraków, Department of Plant Physiology, ul. Podłużna 3, 30-239 Kraków, Poland**,** Email: rrrapacz@cyf-kr.edu.pl

Table S2. Primers of 31 SSRs identified in distance of ± 5cM to DArT markers located in 9 regions affecting drought tolerance of barley and annealing temperatures (Ta) used in PCR reaction.

| Marker | T_a_ (°C) | Primer sequence (5’-3’) | | References |
| --- | --- | --- | --- | --- |
| Bmac047 | 55 | AACACACGTACACAAATACACA | ACGTCCATCACTTTGACC | Ramsay et al. (2000) |
| Bmac144 | 58 | ACTTATTCTGCATCCTGGGT | TACGTGTACATACTCTACGATTTG | Ramsay et al. (2000) |
| Bmac209 | 55 | ATGCCTGTGTGTGGACCAT | CTAGCAACTTCCCAACCGAC | Ramsay et al. (2000) |
| Bmag003 | 52 | GATCAAAGAGAACATGCGAT | GTAGTTCAGCATAGACCTACAGG | Ramsay et al. (2000) |
| Bmag006 | 58 | TGCAGTTACTATCGCTGATTTAGC | TTAAACCCCCCCCCTCTAG | Ramsay et al. (2000) |
| Bmag007 | 55 | TCCCCTATTATAGTGACGGTGTG | TGAAGGAAGAATAAACAACCAACA | Ramsay et al. (2000) |
| Bmag131 | 50 | CCTCCACACAAAAAATCC | TTTCAGAAACGGAGTTTTG | Ramsay et al. (2000) |
| Bmag136 | 52 | GTACGCTTTCAAACCTGG | GTAGGAGGAAGAATAAGGAGG | Ramsay et al. (2000) |
| Bmag206 | 55 | TAGAACTGGGTATTTCCTTGA | TTTTCCCCTATTATAGTGACG | Ramsay et al. (2000) |
| Bmag210 | 52 | ACCTACAGTTCAATAGCTAGTACC | GCACAAAACGATTACATCATA | Varshney et al. (2007) |
| Bmag211 | 52 | ACATCATGTCGATCAAAGC | ATTCATCGATCTTGTATTAGTCC | Sato et al. (2009) |
| Bmag378 | 55 | ATCCAACTATAGTAGCAAAGCC | CTTTTGTTTCCGTAGCATCTA | Ramsay et al. (2000) |
| Bmag500 | 55 | AATGTAAGGGAGTGTCCATAG | GGGAACTTGCTAATGAAGAG | Ramsay et al. (2000) |
| Bmag603 | 52 | ATACCATGATACATCACATCG | GGGGGTATGTACGACTAACTA | Ramsay et al. (2000) |
| Bmag613 | 55 | AAGAACACCATATGATCCAAC | CTCCATGACTATGAGGAGAAG | Ramsay et al. (2000) |
| Bmag692 | 52 | GCAAGGTATCTCTTGTATTTTG | TGGCATCTACAATCTAAAACA | Ramsay et al. (2000) |
| Bmag0867 | 55 | CCCCACACTGACCTACAG | TTACATCTGCTAGATCGAAGC | Hayden et al. (2008) |
| Bmag872 | 52 | ATGTACCATTACGCATCCA | GAAATGTAGAGATGGCACTTG | Ramsay et al. (2000) |
| Bmag876 | 55 | AATTAAAAGCTGAAGGTCTACA | CTGCTCCTTCAACGACTAC | Hayden et al. (2008) |
| EBmac405 | 55 | ATGTAGCTCGGAATGTGTAGT | CATGTTGGATAAGAGTAGAGGA | Varshney et al. (2007) |
| EBmac602 | 55 | CCGTCTAGGGAGAGGTTCTC | GATTGGAGCTTCGGATCAC | Varshney et al. (2007) |
| EBmac713 | 52 | GGTAAAACATTTCCCTCGT | TAGAGATCACTCTCTTCTGTGC | Ramsay et al. (2000) |
| EBmag794 | 55 | CAGTCATAACCTGATGAACAA | TCACACTTATCTTGCTGCTAA | Ramsay et al. (2000) |
| GBM1208 | 58 | CTACCGAGCTCCTCCTCCTC | GGCCTCCTTCTTGTCGTAGA | Varshney et al. (2007) |
| GBM1400 | 55 | AGCGCTCTCTCACACACAGA | ATTCCTGCCTGTTTTTCGTG | Varshney et al. (2007) |
| GBMS180 | 58 | GGAACTAATGCTTCGGTCCA | TGGTGCAAGTGAGCACCTAC | Li et al. (2003) |
| HvLOX | 52 | CACCCTTATTTATTGCCTTAA | CAGCATATCCATCTGATCTG | Varshney et al. (2007) |
| HVM04 | 58 | AGAGCAACTACCAGTCCAATGGCA | GTCGAAGGAGAAGCGGCCCTGGTA | Saghai Maroof et al. (1994) |
| HVM44 | 58 | AAATCTCAGGTTCGTGGGCA | CCACGGAGACCACCTCACTT | Liou et al. (1996) |
| scssr02093 | 55 | CGTCACGCACACATCGAC | GATCTCTCCTCGGGCATC | Varshney et al. (2007) |
| scssr02503 | 55 | AACAACTTTTGATGGACAAACC | TGTCTTTTCTTTTTGCTCTGC | Szűcs et al. (2009) |

Hayden MJ, Nguyen TM, Waterman A, Chalmers KJ (2008) Multiplex-Ready PCR: A new method for multiplexed SSR and SNP genotyping. BMC Genomics 9: 80.

Li JZ, Sjakste TG, Rӧder MS, Ganal MW (2003) Development and genetic mapping of 127 new microsatellite markers in barley. Theor Appl Genet 107:1021–1027

Liou ZW, Biyashev RM, Saghai Maroof MA (1996) Development of simple sequence repeat DNA markers and their integration into a barley linkage map. Theor Appl Genet 93: 869-876

Ramsay L, Macaulay M, degli Ivanissevich S et al. (2000) A simple sequence repeat-based linkage map of barley. Genetics 156: 1997-2005

Saghai Maroof MA, Biyashev,RM, Yang GP, Zhang Q, Allard RW (1994) Extraordinarily polymorphic microsatellite DNA in barley: species diversity, chromosomal locations, and population dynamics. Proc Natl Acad Sci USA 91: 5466-5470

Sato K, Nankaku N, Takeda K (2009) A high-density transcript linkage map of barley derived from a single population. Heredity 103: 110-117

Szűcs P, Blake VC, Bhat PR, Chao S, Close TJ, Cuesta-Marcos A, Muehlbauer GJ, Ramsay L, Waugh R, Hayes PM (2009) An Integrated Resource for Barley Linkage Map and Malting Quality QTL Alignment. The Plant Genome 2: 134-140

Varshney RK, Marcel TC, Ramsay L et al. (2007) A high density barley microsatellite consensus map with 775 SSR loci. Theor Appl Genet 114: 1091-1103
